# Acetylation of alginate enables the production of inks that mimic the chemical properties of *P. aeruginosa* biofilm†

**DOI:** 10.1039/d4tb02675f

**Published:** 2025-01-22

**Authors:** Stephan Schandl, Goodness Osondu-Chuka, Giuseppe Guagliano, Stjepan Perak, Paola Petrini, Francesco Briatico-Vangosa, Erik Reimhult, Olivier Guillaume

**Affiliations:** a Institute of Materials Science and Technology, Technische Universität Wien Vienna Austria stephan.schandl@tuwien.ac.at; b Austrian Cluster of Tissue Regeneration Austria https://www.tissue-regeneration.at; c Department of Bionanosciences, Institute of Colloid and Biointerface Science, BOKU University Austria; d Department of Chemistry, Materials and Chemical Engineering ‘G Natta’, Politecnico di Milano Milan Italy

## Abstract

The reason why certain bacteria, *e.g.*, *Pseudomonas aeruginosa* (PA), produce acetylated alginate (Alg) in their biofilms remains one of the most intriguing facts in microbiology. Being the main structural component of the secreted biofilm, like the one formed in the lungs of cystic fibrosis (CF) patients, Alg plays a crucial role in protecting the bacteria from environmental stress and potential threats. Nonetheless, to investigate the PA biofilm environment and its lack of susceptibility to antibiotic treatment, the currently developed *in vitro* biofilm models use native seaweed Alg, which is a non-acetylated Alg. The role of the acetyl side group on the backbone of bacterial Alg has never been elucidated, and the transposition of experimental results obtained from such systems to clinical conditions (*e.g.*, to treat CF-infection) may be hazardous. We systematically investigated the influence of acetylation on the physico-chemical and mechanical properties of Alg in solution and Ca^2+^-crosslinked hydrogels. Furthermore, we assessed how the acetylation influenced the interaction of Alg with tobramycin, a common aminoglycoside antibiotic for PA. Our study revealed that the degree of acetylation directly impacts the viscosity and Young's Modulus of Alg in a pH-dependent manner. Acetylation increased the mesh size in biofilm-like Alg hydrogels, directly influencing antibiotic penetration. Our results provide essential insights to create more clinically relevant *in vitro* infection models to test the efficacy of new drugs or to better understand the 3D microenvironment of PA biofilms.

## Abstract 2

Seaweed-derived alginate was acetylated to mimic the degree of acetylation of *P. aeruginosa* alginate. Inkjet printing was used to produce microbeads as 3D biofilm models to investigate the influence of the acetylation.

## Graphical abstract

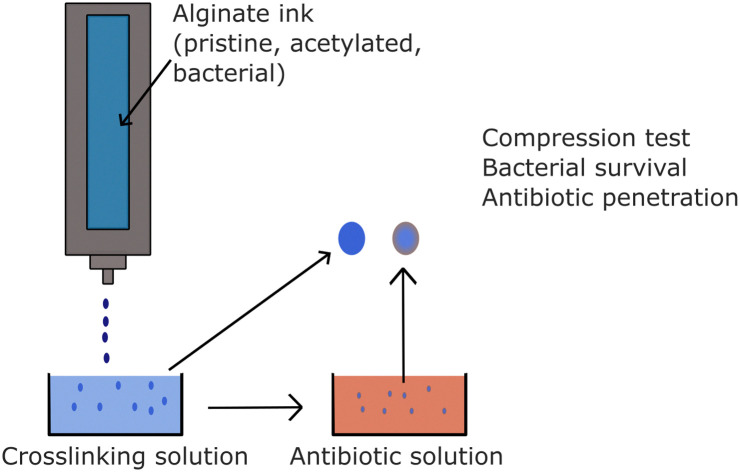

## Introduction

Bacterial biofilms are an essential field of research due to their complexity and omnipresence in nature.^1^ Their presence on surfaces of pipelines in the sewage system,^2,3^ on building surfaces,^4–6^ or in the tubings of catheters in hospitals^7–9^ calls for efficient strategies to eradicate biofilms. This is especially true for patients suffering from CF, who are at high risk of developing bacterial infections, leading to biofilm formation in the mucus layer of their airways.^10^ CF leads to an osmotic imbalance, causing dehydration and thickening of the mucus-rich layer on top of the epithelial lung cells.^11^ This highly viscous environment is fertile ground for bacteria, like *Pseudomonas aeruginosa* (PA) or *Staphylococcus aureus*, to spread. Infections from PA, a strong biofilm producer, often lead to complications in antibiotic treatment as the bacteria can switch their phenotype from an initial wild type to a mucoid type.^10^

Once this switch occurs, the Alg production of PA is highly upregulated. Alg is exported to the extracellular matrix of biofilms, where it plays a central role in the mucoid biofilm.^12^ It significantly enhances bacterial biofilms’ structural and functional integrity through its gelation ability, forming stable gels with divalent cations like calcium.^12^ These viscoelastic gels’ chemical and physical stability allows the bacteria community to withstand mechanical stresses while maintaining flexibility. The hydrophilic nature of Alg ensures high water retention and porosity, which are critical for nutrient and waste transport within the biofilm. The high molecular weight Alg, together with other components of the extracellular polymeric substances (EPS),^13^ also protects the bacterial colonies from environmental and chemical threats, *e.g.*, by limiting diffusive transport and binding toxic compounds such as antibiotics.^14–16^

Alg consists of β-d-mannuronic (M) acid and α-l-guluronic (G) acid units. The G units are responsible for gelation by crosslinking *via* divalent cations, mainly Ca^2+^, while blocks of M units are assumed to have higher hydration and flexibility. One of the intriguing aspects regarding biofilm Alg from PA is that, unlike seaweed-derived Alg, it is modified with acetyl side groups on the mannuronic acid block (M).^17^ The remaining guluronic acid block (G) is not modified, retaining the gelation properties.^17,18^ The reason why bacteria are acetylating the Alg and how this influences the properties of the biofilm is not fully understood. Previous studies showed that some properties, *e.g.*, the viscosity of Alg solutions^19^ or metal-induced precipitation, can be tuned by the degree of acetylation.^18,20^ It was also proposed that acetylation leads to higher water uptake, potentially protecting the bacteria from dehydration and increasing bacteria adhesion within the biofilm.^18^

Current *in vitro* CF biofilm models use seaweed derived Alg.^14,21,22^ or natively generated biofilms directly from cultured PA.^23,24^ Models using native PA biofilm suffer from time-consuming preparations and inhomogeneities within the biofilm samples.^25^ Furthermore, natural biofilms contain not only Alg but other components, *e.g.*, extracellular DNA^12^ and other polymeric substances.^13^ Designing reliable *in vitro* models of biofilm environments is necessary to decipher each compound's contribution to, for instance, the acquisition of antibiotic tolerance due to drug–EPS interaction.^13,26–28^ Hence, producing Alg starting from seaweed with a controlled degree of acetylation would be advantageous.

Inkjet printing is an easy to use biofabrication method as it allow direct encapsulation of cells,^29^ protein and other biomolecules.^30^ Despite the variety of inkjet-based printing methods, from pneumatically to electrostatically and thermally driven jetting, they all rely on relatively low viscosity inks.^31^ (Bio)inks for inkjet printing allow the controlled deposition and encapsulation in droplets down to fl in volume.^31^ Using Alg as ink leads to the fabrication of droplets, in which cells can be encapsulated for 3D cell culture^29,32^ but also (bio)molecules can be encapsulated to use Alg microgels as delivery vehicles.^29^ Although 3D bioprinting is widely used in biofabrication in context with mammalian cells, bioprinting with bacteria is still in development. Inkjet printing can be used to produce microgels in a high-throughput fashion with adjustable sizes to mimic biofilms in all stages. This was illustrated by Ning *et al.* who showed that extrusion-based bioprinting was an excellent method for the production of *in vitro* biofilm models of thickness between 0.25 and 4 mm.^33^ Inkjet printing would allow to produce microgels in a high-throughput fashion to biofilm models with adjustable sizes to mimic biofilms in all stages. Nevertheless, as any other reports of *in vitro* CF-like biofilms, they use seaweed-derived alginate for building those biofilms, omitting the acetyl group of the alginate.

In this study, we investigate the influence of acetylation on the physical and mechanical properties of Alg solutions and Ca^2+^-crosslinked hydrogels, respectively. We first developed a protocol to optimize the degree of acetylation of seaweed Alg. Then, we addressed the influence of the pH of the surrounding media on the mechanical properties of acetylated Alg. This was performed to mimic acidic conditions clinically observed during the maturation process of CF-biofilms. Finally, the interaction between tobramycin and Alg and the influence of its acetylation were investigated from the molecular to the macroscopic level.

## Materials and methods

### Materials

Sodium Alg Pronova-UP-MVM (60% M content) was obtained from Novamatrix FMC Biopolymer (Sandvika, Norway). All Alg was used as sodium salts and will be referred to as Alg. Pyridine, acetic anhydride, tobramycin sulphate (potency 0.699 mg mg^−1^), disodium ethylenediaminetetraacetic acid (EDTA), fluorescein isothiocyanate dextran (FITC-dextran, 70, 150, 500 and 2000 kDa dextran size), CaCl_2_–2H_2_O, *ortho*-phthaldialdehyde reagent and 2-(*N*-morpholino)ethanesulfonic acid (MES) hydrate were obtained from Sigma-Aldrich. Pyridine was dried over a 4 Å molecular sieve (Sigma-Aldrich, Vienna, Austria) and always kept under N_2_ atmosphere. Ultrapure water was provided by an in-house water purification system (ELGA Chorus, ELGA LabWater, Germany). All Alg solutions were made using ultrapure water unless stated otherwise. *Pseudomonas aeruginosa* (39324™) was obtained from ATCC®. LB medium and *Pseudomonas* isolation agar were obtained from Carl Roth and Sigma Aldrich, respectively.

### Methods

#### Alg acetylation

The Alg acetylation was performed according to a modified procedure from literature.^34^ Alg beads were prepared by dropping 1 wt% Alg solution into 0.1 M CaCl_2_ solution. The beads were left in the crosslinking bath for 24 h before exchanging the solvent from water to pyridine. Pyridine was exchanged after 3 and 24 h to ensure complete dehydration of the Alg beads. The medium was then exchanged to pyridine with Ac_2_O, with a total volume of 100 ml g_Alg_^−1^. The volume of pyridine was adjusted with Ac_2_O volume to obtain the exact concentration throughout all tests. The reaction was stirred for 24 h at room temperature, then filtered off the beads and washed twice with acetone and deionized water (100 ml per washing step with 10 min stirring in between). The beads were dissolved overnight in 50 ml 0.2 M EDTA (pH 7.4) before dialysis (molecular weight cut-off 3.5 kDa, Sigma-Aldrich) against 0.9% NaCl for 48 h, followed by deionized water for 48 h. After dialysis, the solution was concentrated to approximately half the volume using a rotary evaporator before lyophilization (Christ Gamma 2–20, Osterode am Harz, Germany). The product was obtained as a cotton-like material and stored at −20 °C under an N_2_-atmosphere.

### Bacterial Alg isolation

Bacterial Alg was isolated from native *Pseudomonas aeruginosa* (39324™) biofilms grown on agar plates according to a procedure adapted from literature.^35^ An overnight culture of PA in LB medium was harvested by centrifugation. After resuspending the bacteria, 200 μl were spread onto *Pseudomonas* isolation agar and incubated at 37 °C for 72 h. The formed biomass was scraped off and suspended in a saline solution. The suspension was centrifuged at 25 000*g*. The supernatant was precipitated in ice-cold 2-propanol and left to precipitate overnight at 4 °C. The precipitate was collected by centrifugation at 15 000*g* and thoroughly washed three times with ice-cold 2-propanol. Following resuspending in 50 mM Tris–HCl with 1 mM CaCl_2_ and 2 mM MgCl_2_ (pH 7.5), 15 μg ml^−1^ DNase I and 15 μg ml^−1^ RNase A were added, and the solution incubated at 37 °C for 6 h to remove nucleic acids. 5 mg ml^−1^ Proteinase K was added, and the solution was incubated at 37 °C for 24 h to digest proteins before dialyzing the solution (molecular weight cut-off 14 kDa, Carl Roth) against 5 l ultrapure H_2_O. The solution was freeze-dried, and bacterial Alg was obtained as an off-white cotton-like material. The concentration of alginate in solution was determined by a modified carbazole method,^36^ in which a solution of the purified alginate (50 μl, 1 mg ml^−1^) was mixed with 200 μl of borate–sulfuric acid reagent (10 mM H_3_BO_3_ in concentrated H_2_SO_4_) and 50 μl of carbazole reagent (0.1% in ethanol). The mixture was then incubated at 100 °C for 10 min, and absorbance at 550 nm was determined spectrophotometrically. The alginate concentration was determined by interpolation from a standard curve (0 to 1 mg ml^−1^) of alginic acid from seaweed (Pronova UP-MVM, Novamatrix FMC Biopolymer). For each sample or dilution, a negative control was assayed by using 0.0125 M NaOH.

### Nuclear magnetic resonance (NMR) spectroscopy

^1^H-NMR spectra of Alg solutions were recorded on a BRUKER Avance DRX-400 FT-NMR spectrometer. Chemical shifts were indicated in ppm relative to trimethylsilyl propanoic acid (*δ* = 0 ppm) and referenced on the used NMR solvent. Obtained spectra were analyzed using the MestReNova software by Mestrelab Research. Before ^1^H-NMR, samples were treated with Alg lyase according to the manufacturer's protocol for 24 h, followed by precipitation in EtOH. After drying at 60 °C, the pellet was dissolved in deuterated water. The degree of acetylation (d. ac.) was calculated as described in the literature^17^ using the following formula:
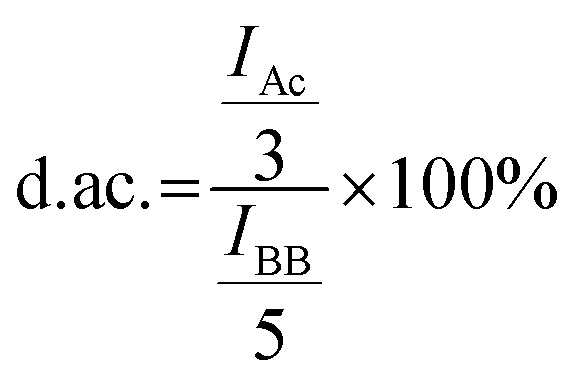
with d. ac., *I*_Ac_, and *I*_BB_ being the degree of acetylation, the integral of the acetyl peak, and the integral of the Alg backbone (up and downfield of the solvent peak), respectively. The factors 3 and 5 are derived from the three protons per acetyl side group and five protons per monomeric unit.

### Gel permeation chromatography (GPC)

Gel permeation chromatography was performed on a Viscotek GPC system, including a Viscotek GPCmax VE2001 GPC solvent/sample module (100 μl loop), a solvent degasser, a Viscotek VE3580 refractive index concentration detector. A Waters pre-column, a Waters ultrahydrogel linear 7.8 × 30 mm column and a Waters ultrahydrogel 250 7.8 × 30 mm column with 10 μm particle size were used for separation. A PBS buffer (NaH_2_PO_4_ 5 mM, NaCl 100 mM. NaN_3_ 0.05%, pH 7.4) was used as eluent at 30 °C and a flow rate of 1 ml min^−1^. Pullulan calibration standards (Shodex Standard P-82) were used for standard calibration to determine the number average molecular weight (
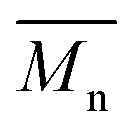
) and polydispersity index (PDI). The samples were prepared by dissolving the sodium Alg in a concentration of 2 mg ml^−1^ in PBS buffer and syringe filtered before measurements.

### Preparation of Alg solutions

Unless otherwise stated, stock solutions of Alg with a concentration of 69 mM were prepared by dissolving the appropriate weight of Alg in ultrapure water under slow agitation overnight. The stock solutions were then diluted to the desired concentrations stated in each section using ultrapure water.

### Inkjet printing of Alg beads and drug-induced shrinkage test

A 23 mM solution of Alg was loaded into a cartridge for a pneumatic pump, inserted into an EMD printhead (Cellink, BICO, Sweden), and mounted on a BioX Gen3 (Cellink, BICO, Sweden). The pressure applied to the formulation for inkjet printing ranged from 45 to 90 kPa, depending on the type of Alg. The printing parameters for open and cycle time and writing speed were set to 1 and 150 ms and 1.5 mm s^−1^, respectively. The formulation was jetted into the wells of a 48-well plate containing 800 μl of a sterile, filtered 350 mM CaCl_2_ solution per well. The produced microbeads were left to crosslink in the solution overnight. Sample groups for drug-induced shrinkage were washed twice with deionized water prior to incubation with a 0.7 mg ml^−1^ tobramycin solution in 10 mM MES buffer (pH 6.5). Images of the microbeads were taken at regular intervals, depending on the speed of the shrinkage, using a stereomicroscope (Evident, Germany) in darkfield mode to visualize the microbead's perimeter better. The printing was performed into one well with at least 100 beads. Images were taken of the entire well, and image analysis was performed using the Fiji ImageJ package.^37^ To analyze the tobramycin uptake, 85 μl Alg solution were transferred into an agarose mold with 100 mM CaCl_2_ and left to crosslink overnight. The prepared gels were preconditioned in 10 mM MES buffer (pH 6.5) overnight before incubation in 0.7 mg ml^−1^ tobramycin solution. The gels were then dissolved in 150 mM EDTA solution (pH 7.4) for 30 min under rotation, and tobramycin quantified using *o*-phthaldialdehyde reaction according to a reported protocol.^38^ The amount of tobramycin was then normalized against the weight of each Alg hydrogel.

### Ink and hydrogel characterizations

The viscosity of Alg solutions was analyzed on an Anton Paar MCR 300 device with a P-PTD 200/GL Peltier glass plate and a CP25 measuring system (Anton Paar, Graz, Austria). The plate was heated to 25 °C, and 85 μl of formulation was added. The measuring gap size and shear rate were set to 0.048 mm and 100 rad s^−1^, respectively. After 45 s of equilibration, 5 measurement points were recorded in succession, and the mean was taken as viscosity in mPa s. The formulations were tested in technical triplicates with duplicate measurements for each formulation.

Oscillation mode measurements were performed on an Anton-Paar MCR 502 (Anton Paar, Graz, Austria) with a 25 mm plate-plate geometry at 25 °C. The gels were prepared according to literature, by exploiting an internal gelation process.^21,39^ In short, a 0.7 wt% solution of Alg was mixed with a 1 wt% suspension of CaCO_3_ and a 1.4 wt% solution of glucono-*δ*-lactone in a 5 : 1 : 1 ratio. 250 μl samples were pipetted into Petri dishes and left to crosslink overnight. For the testing of different media, the gels were either measured as they were, at pH 7, or incubated in 10 mM MES buffer at pH 6.5 for 3 h before the measurements. The linear viscoelastic region (LVER) of Alg hydrogels was determined by strain sweeps ranging from 0.01% to 100% at an oscillatory frequency of 1 s^−1^. The yield strain was defined as the intersection of storage (*G*′) and loss modulus (*G*′′), indicating the transition from solid-like to liquid-like behaviour. The LVER was determined using the built-in analysis tool in RheoCompass 1.13 (Anton Paar, Graz, Austria) and defined as the region until *G*′ leaves a tolerance band of 5%. Frequency sweeps ranged from 0.314 to 125 rad s^−1^ at a strain of 0.03%, and the microstructure was modelled according to literature by interpreting the response in frequency with the generalized Maxwell model.^40^

The densities of Alg solutions were determined by employing pycnometry. The volume of the pycnometer was approximately 1 ml. The pycnometer and stock solutions of tested Alg samples were kept in a water bath at 20 °C for 2 h for thermal equilibration. The pycnometer, approximately 1 ml in volume, was calibrated with water, and the density of each solution was determined based on the mean value of triplicates.

The surface tension of Alg solutions was measured using the pendant drop method on a Krüss DSA30 device according to the manufacturer's protocol. In short, a drop was extruded on the tip of a 1.8 mm needle, and the contour fitted at maximum size before the drop detached from the tip.

### Mechanical microtesting

Compression tests were performed on a MicroTester (Cellscale, Canada) in compression mode. The sample specimens were prepared as described above and transferred onto the sample holder using 1 ml syringes without needles. The microbeads were washed twice with 1 ml ultrapure water to test different pHs and then incubated in 10 mM MES buffer at a given pH with 3 mM CaCl_2_ to mimic Ca^2+^ levels in the CF sputum.^26,27^ Next, the force loads were recorded as a function of deformation. The Young's modulus of the microspheres was determined using the Hertz formula:^41^
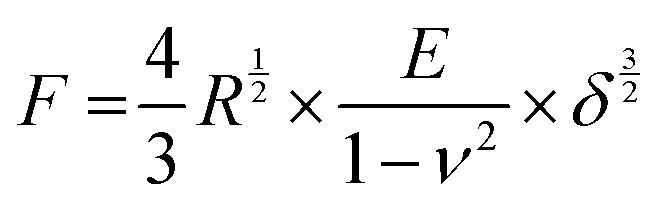
with *F*, *R*, *E*, *ν* and *δ* being the recorded force, the radius of the microbeads, the Young's modulus, the Poisson coefficient and the displacement, respectively. The Poisson coefficient was assumed to 0.5, according to literature.^42^

### Fluorescence recovery after photobleaching (FRAP)

FRAP experiments were performed on a confocal laser scanning microscope (Zeiss CLSM800, Carl Zeiss, Germany) with a 20× magnification objective. Hydrogel disks were prepared by external gelation in an agarose mould. For this, 100 μl of Alg solution were filled into the agarose mold (2 wt% agarose, 100 mM CaCl_2_) and left to crosslink overnight. The disks were then incubated in 10 mM MES buffer at the chosen pH with 3 mM CaCl_2_ and 1 μM FITC-dextran overnight. Before performing FRAP experiments, the Alg hydrogel disks were dried on a paper towel. Three different FITC-dextran sizes (70, 150, and 500 kDa) were used as molecular probes to investigate the diffusion coefficient within the gel. A literature-reported automated batch evaluation of FRAP experiments in hydrogels was used to determine the diffusion coefficients.^43^ The hydrogel pore radii were then calculated from the obtained diffusion coefficients by the formula:^44,45^
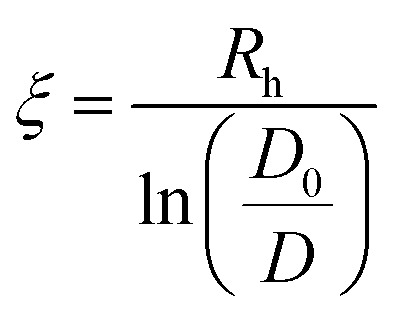
with *ξ*, *R*_h_, *D*_0_, and *D* being the pore radius, the hydrodynamic radius, and the diffusion coefficient in unrestricted and restricted environments, respectively.

### Isothermal titration calorimetry (ITC)

Isothermal titration calorimetry was performed on an automated PEAQ-ITC (Malvern Panalytica, UK) at 25 °C in high feedback mode and a reference power of 5 μcal s^−1^. 0.1 wt% stock solutions of Alg were dialyzed against a 10 mM MES buffer (pH 6.5) overnight and exchanging the dialysis buffer after 4 h. The dialysis buffer was used to dilute the samples to a final concentration of 0.01 wt% and to prepare a 10 mM tobramycin stock solution. The titration was performed in 19 steps, with the first injection being 0.2 μl followed by 18 2-μl injections of tobramycin into Alg. The analysis of the thermogram was performed using NITPIC^46^ and SEDPHAT.^47^ Plotting of the integrated ITC thermogram was performed using GUSSI.^48^

### Data analysis and statistical analysis

Data plotting and statistical analysis were performed using OriginPro 2022 (Origin Lab). All results are depicted as mean ± standard deviation (SD). Tukey tests were performed to assess the statistical significance between the sample groups. A *p* < 0.05 was considered of statistical significance.

## Results and discussion

### Optimization of the chemical acetylation protocol

In their native biofilm form, mucoid PA secrete acetylated Alg as a major matrix component. This acetylated Alg has been analyzed to have a degree of acetylation (d. ac.) of 22–40%, depending on the strain and maturity of the biofilm.^17^ It is important to synthetically control the degree of acetylation to develop a CF-like biofilm environment starting from seaweed Alg. The literature reports two distinct ways of synthesizing such Alg.^49^ One method is unspecific acetylation of all available hydroxyl groups on the backbone, while the other specifically protects homopolymeric guluronic acid (G) blocks with di- or multivalent cations.^18,50,51^

**Scheme 1:**
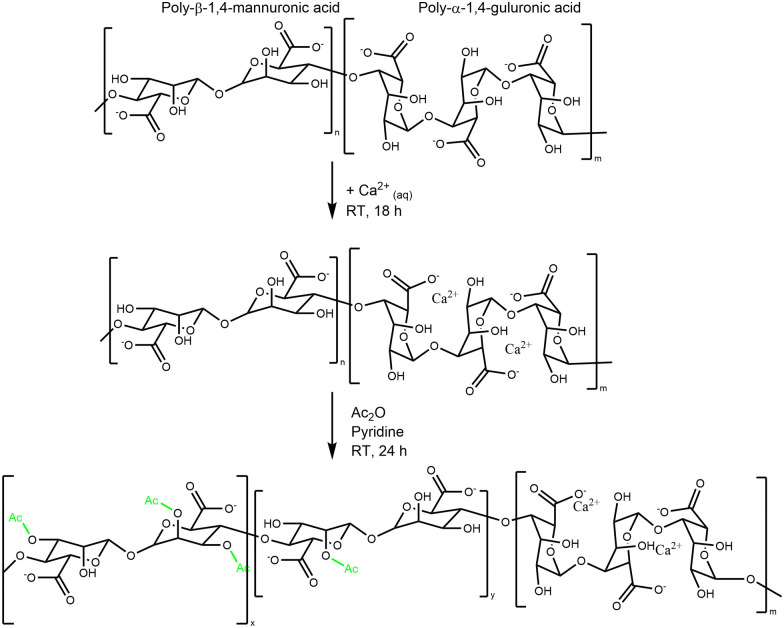
Generalized flow scheme of the chemical acetylation of Ca–Alg hydrogels.

In the unspecific acetylation approach, Alg is first solubilized in organic solvents by completely protonating the uronic acid or using organic quaternary ammonium ions as counterions.^52^ It was reported that such global acetylation of the Alg backbone reduced Alg solubility, and the resulting product lost its ability to form Ca^2+^ crosslinked hydrogels.^18,20^ In contrast, selective acetylation exploits the specific interactions between the G-block and metal ions (*e.g.*, Ca^2+^).^34^ Hydrogel beads are formed by dropping the Alg solution into a crosslinker bath, usually a 100 mM CaCl_2_ solution. The Ca^2+^ ions sterically occupy the G-blocks; consequently, only the mannuronic acid (M) blocks are available for modificiation (*e.g.* to react with Ac_2_O). This results in the synthesis of an Alg, mostly acetylated on its M units, like the one produced from PA.^34^ The global synthesis reaction is shown in Scheme 1.^18,19,52^

**Fig. 1:**
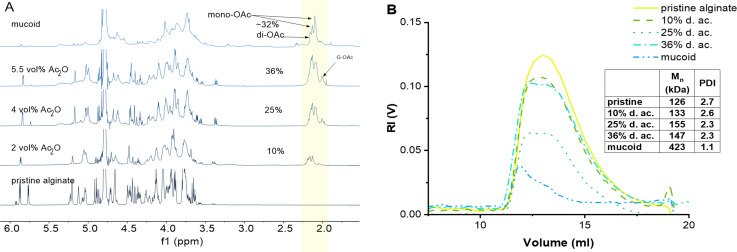
(A) ^1^H-NMR comparison between chemically acetylated Alg as a function of Ac_2_O vol%, and native Alg isolated from mucoid PA biofilm. The peak of the acetyl side group (2.02–2.2 ppm, highlighted area) increases with the increasing concentration of used Ac_2_O. (B) GPC of pristine, modified, and mucoid Alg.

We found that the amount of Ac_2_O can be effectively reduced from typically 100 ml^34^ Ac_2_O down to 5.5 ml Ac_2_O per g of Alg and increases the control of the d. ac. by varying the molar ratios between Alg and Ac_2_O (Fig. 1(A)). We could also demonstrate that drying pyridine over molecular sieves reduces the solvent's moisture content and increases the efficiency of Ac_2_O (see SI 2.2., ESI†).

Importantly, we were able to show that by simply varying the concentrations of Ac_2_O (2, 4, and 5.5 vol%), it is possible to control the degree of acetylation on the Alg chain while keeping the reaction time constant. The ^1^H-NMR spectra clearly indicate that the peak of the acetyl side group at 2.0–2.2 ppm increases as a function of Ac_2_O concentration (Fig. 1(A))

Our protocol enabled us to control the acetylation of Alg by adjusting the concentration of Ac_2_O, which has not been reported yet in the literature to the best of our knowledge.

Using this protocol, the reaction was optimized to obtain a range of degrees of acetylation that covers the ones reported to be secreted by PA.^17^ One limitation of this protocol, which is visible in the acetylation pattern, is the relatively high amount of di-acetylated M units, which is not present in the mucoid Alg. However, we are not aware of a direct way to achieve regiospecific acetylation of either mono- or disubstitution. Acetylation in one position might even activate the monomeric unit to undergo acetylation in the second position due to H-bond accepting features of the acetyl group. Furthermore, the current state-of-the-art does not provide a clear explanation of how the acetylation pattern might influence the properties of Alg chains. Another limitation of synthetical acetylation is the side-reaction of unprotected G units. Indeed, using high concentrations of Ac_2_O leads to acetylation of the G unit located beside M, as indicated by the peak at 1.99 ppm. The increasing amount of free AcO^−^ ions might interact with Ca^2+^ ions as well and chelate enough to weaken the Alg eggbox and allow G units to react with Ac_2_O.

Evans and Linker have reported that PA, isolated from CF patients, secretes a high molecular weight Alg of approximately 360 kDa.^53^ The M_W_ of Alg is crucial for its properties; this is why it is important to develop a chemical protocol that enables not only the acetylation of the seaweed Alg but also maintains the high molecular weight of the starting material.^17^ GPC confirmed that the reaction conditions do not lead to a degradation of the Alg backbone (Fig. 1(B)).^19,34^ Indeed, the *M*_n_ was 170 kDa (PDI 2.7) of the pristine Alg and 190 kDa (PDI 2.3) after acetylation (36% d. ac.). The slight increase in *M*_n_ might be an artifact from a larger coil size measured by GPC caused by the addition of the acetyl side group onto the Alg backbone, leading to increased internal double-layer repulsion between the acetylated chain segments.^54,55^ GPC analysis revealed that Alg isolated from the mucoid PA strain used in this study exhibits an *M*_n_ of 420 kDa (PDI 1.1), which is in accordance with data obtained in the literature using viscosimetric measurements.^53,56^ Although it is unknown to literature how the PDI might influence alginate gel properties, it could affect the viscosity of alginate solutions. The viscosity of polymer solutions is, among other factors, increased with increasing molecular weight of the polymer.^57^

One limitation of our synthesized Alg is that, even though it has a high molecular weight, its *M*_n_ is still half of the one secreted by PA. This shortcoming could be addressed by starting from a higher *M*_n_ Alg than the one we used, but such Algs are, as far as we know, not commercially available with an M/G ratio mimicking that of PA Alg.

### Characterization of acetylated Alg inks and hydrogels

Alg-based biomaterials and bio-inks are generally relatively well characterized in the literature.^58,59^ Alg is frequently added in bio(material) inks for extrusion-based 3D-printing to adjust rheological properties, such as shear thinning and recovery.^39,58,60^ Printed structures can be post-processed with Ca^2+^-induced crosslinking, leading to improved stability of hydrogels. Alg is also used in jetting/spraying-based methods to generate (micro)beads of varying sizes.^30,61^ We used inkjet-printed Alg microbeads as a robust 3D model to produce biofilm-like models to be used to investigate mechanical properties and drug diffusion as a function of d. ac. Those beads can potentially be used to encapsulate dissolved macromolecules, such as DNA, cells, or bacteria.^30,41,61^

**Fig. 2:**
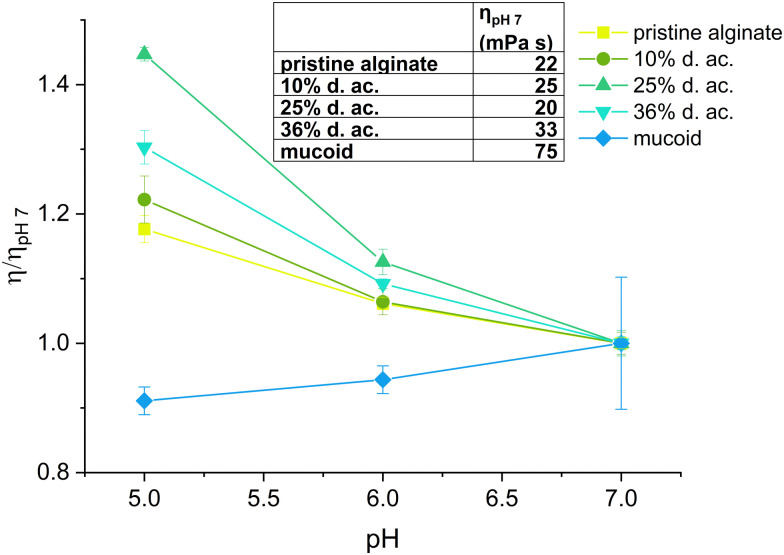
The viscosity of the Alg inks as a function of pH relative to their viscosity at pH 7.

A low ink viscosity is required for inkjet fabrication. Hence, we optimized the ink jet printing by testing pristine Alg at various concentrations, targeting as low concentrations of Alg as possible to reduce the formulation's viscosity. A concentration of 23 mM was established as optimal in the preliminary screening experiments since it allowed the production of high-quality microbeads by inkjet printing. Therefore, we tested all Alg solutions (pristine, modified, and native mucoid) at a fixed molar concentration of 23 mM for their viscosity. The viscosity of the inks was tested at pH 5, 6, and 7, as it covers the pH measured in the CF-biofilm of PA.^62,63^ The results of the viscosity measurements under these conditions are shown in Fig. 2. It was observed that the viscosity first decreased with increasing d. ac., up to 25%, then rose again at 36%, in agreement with previous literature.^19^ This trend was observed for all pHs. The decrease of viscosity from the Alg with low to moderate degrees of acetylation (up to ∼25%) was assumed to derive from a decrease in the interaction between the chains due to the acetylation.^19^ It is speculated that the acetylation leads to a change in the conformation of the polymer in solution, which leads to larger radii of gyration and a reduction in aggregate formation.^20,54^ Reducing the aggregation tendency could explain the viscosity reduction in low to moderate d. ac. (up to 25%). The increased coil size overtakes this trend for higher degrees of acetylation.

**Scheme 2:**
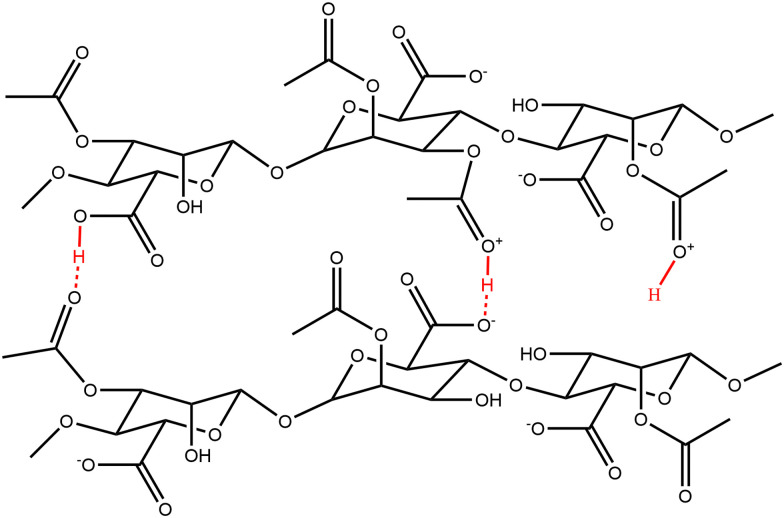
Potential interactions and intercalation positions where protons might interact with the backbone of acetylated Alg. Those positions are not available for native seaweed Alg and are only observed in acidic conditions. Those interactions might increase inter- and intramolecular interactions, affecting the viscosity.

When looking at the influence of the pH on the viscosity of Alg solutions, we observed that an increase in pH led to a decrease in viscosity. This effect was observed for all acetylated Alg, but more pronounced at higher d. ac. While the viscosity of pristine Alg was statistically significantly increasing, its increase from 22 mPa s (pH 7) to 26 mPa s (pH 5) can be assumed to be of technical insignificance. Literature reports a drastic pH-dependent increase in viscosity in highly acidic conditions (pH < 4),^64^ outside of our investigated range, due to partial protonation of the carboxylic acid groups, resulting in phase separation of the Alg solution.^65^ However, this effect cannot explain the trend we found within the investigated pH range. Therefore, the acetyl side groups must be the reason for the pH sensitivity. We propose that inter- and intramolecular interactions of the acetyl side groups lead to the increase in viscosity (Scheme 2), due to the increased specific volume of the Alg coil caused by acetylation.^54^ Interestingly, the mucoid Alg had the highest viscosity at all conditions, likely due to its higher molecular weight. Nevertheless, the mucoid Alg exhibits a different viscosity pattern compared to the synthetic analogues developed in our study. Indeed, its viscosity increased slightly with increasing pH. Additionally, surface tension and density of the Alg inks were of interest, as these are also important for printability (see ESI†). The surface tension was decreased with the addition of Alg and decreased with degree of acetylation. The surface tension was significantly reduced once the acetyl side-group was introduced but there was no statistically significant difference between the synthetically acetylated Alg. The surface tension reduced from 69.2 ± 2 mN m^−1^ (pristine) to 65.9 ± 0.2 mN m^−1^ (36% d. ac.). Mucoid alg (65.6 ± 0.3 mN m^−1^) showed no statistically significant difference with the Alg with 36% d. ac. The density of the Alg inks varied between 1.0024 g cm^−3^ (mucoid) and 0.9980 g cm^−3^ (36% d. ac.) with no statistically significant difference between the tested Alg. Values for surface tension and density are well in accordance with other reported values for Alg-based inks for inkjet printing.^66,67^ Therefore, we concluded that printability was indeed not affected by the acetylation.

For inkjet production of microbeads as 3D models for bacterial biofilms, we sought to use the lowest viscosity found at pH 7. These microbeads can be produced in a high-throughput, reproducible manner and allow for a wide range of systematic studies, such as antibiotic interactions, mechanical properties, and bacterial encapsulation, which are highly relevant for modelling bacterial biofilms’ physicochemical properties.

**Fig. 3:**
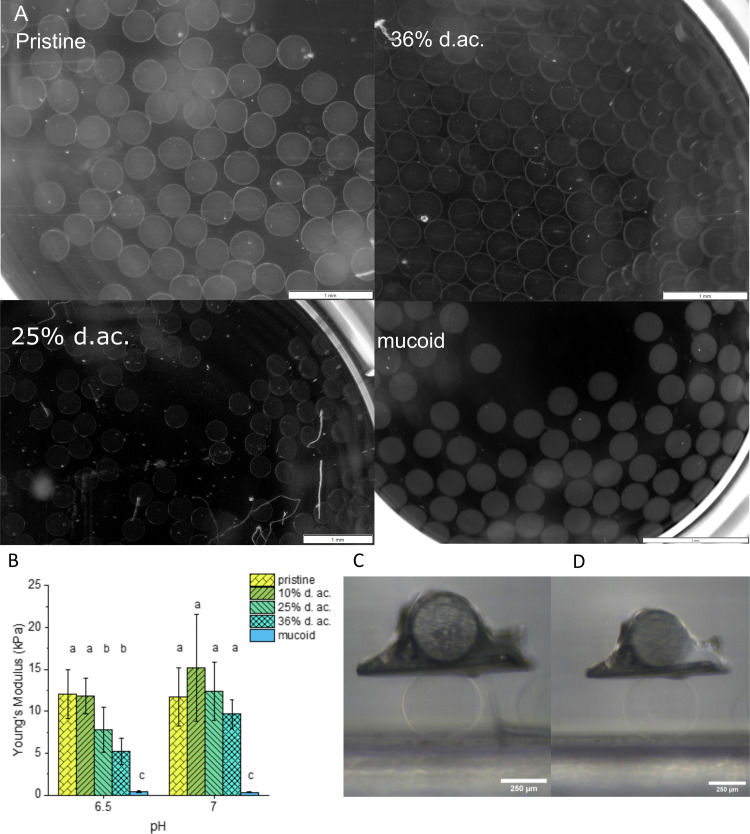
(A) Representative images of printed Alg microbeads after 24 h in 300 mM CaCl_2_ solution (scalebar for pristine, 25% and 36% d. ac. = 1 mm; scalebar for mucoid 2 mm). (B) The Young's Modulus of Alg microbeads with varying degrees of acetylation at pH 7 and 6.5. Microbeads of pristine (C) and 36% d. ac. (D) during the microtesting. Scalebar 250 μm. Different letters indicate statistical significance (*p* < 0.05).

Examples of those microbeads as printed and during mechanical testing are shown in Fig. 3(A), (C) and (D), respectively. The microbeads had radii between 200–240 μm, with no statistically significant difference between the formulations. This confirms that the acetylation does not negatively influence the Ca^2+^-induced crosslinking, following the literature for the investigated range of d. ac.^20^ It has been shown that the Ca^2+^ concentration in the mucus layer of CF patients is elevated and can reach up to 5 mM.^26,27^ These elevated Ca^2+^ concentrations will stiffen the extracellular matrix of PA.^28^ To investigate how the acetylation alters the stiffness of Alg gels, Alg microbeads were tested for their Young's Modulus in compression tests in media at pH 6.5 and 7. These two pHs were chosen to mimic the early stages of PA infection in CF patients. The Alg chains are spatially confined in the gel state, and small pH changes should have a larger impact on mechanical properties than on the viscosity in the solute state. The Young's Moduli (see Fig. 3(B)) at pH 7 ranged between 9.8 and 12 kPa with no significant impact of the acetylation observable. When changing the pH from 7 to 6.5, no change in Young's Modulus was observed for pristine Alg and 10% d. ac. However, for 25% and 36% d. ac., a reduction was observed to 7.5 (−42%) and 5 kPa (−46%), respectively. The reduced stiffness is most likely caused by a reduced affinity of the Alg backbone to Ca^2+^ ions, as shown by Lee *et al.*^68^ Mucoid Alg, which contains almost no consecutive G-units, showed a drastically lower E-Modulus of 0.5 kPa. The lower M/G ratio (>1.5, according to the manufacturer) of the pristine seaweed-derived Alg compared to mucoid Alg could explain this drastic difference, as the poly-G blocks provide mechanical stiffness.^69,70^

We followed two approaches to investigate how acetylation influences the molecular network of Alg, *e.g.*, its mesh size, and if an increase in mesh size from reduced crosslinking causes the reduced Young's moduli. Using rheology allowed us to model a mechanical mesh size, *ξ*_Rheo_, while FRAP allowed us to model a molecular mesh size, *ξ*_FRAP_, which gave insights into how large a solute may be before reptation occurs. Both parameters are helpful for designing drugs or drug delivery systems that need to penetrate bacterial biofilms.

**Fig. 4:**
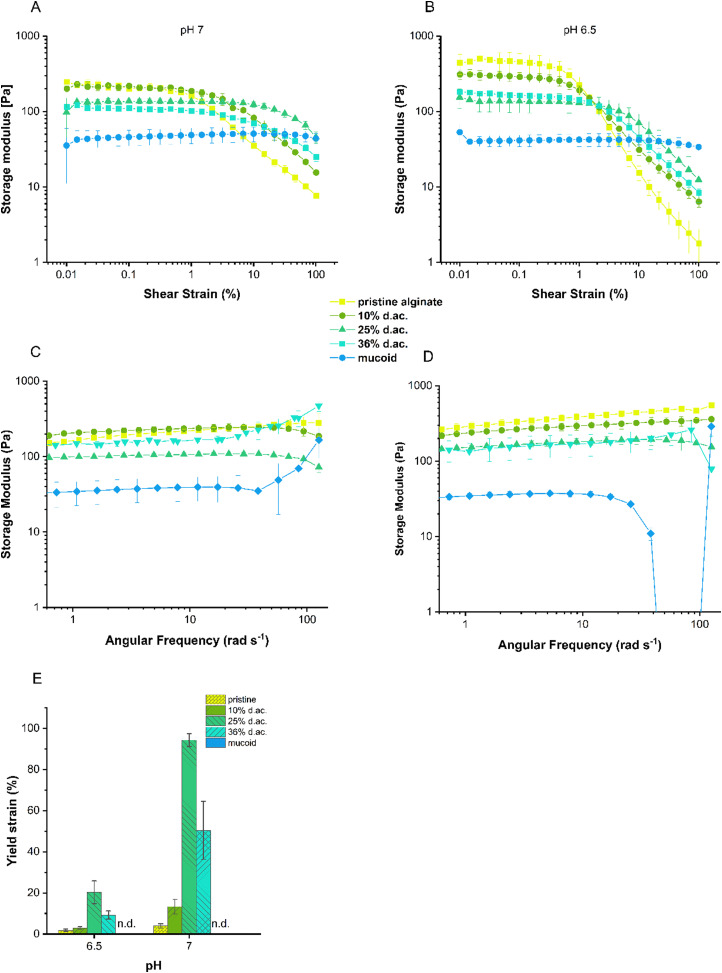
Amplitude sweep of the hydrogels at pH 7 (A) and pH 6.5 (B). The storage modulus obtained from the frequency sweeps, C and D. The yield strain (E) was increased with increasing d. ac. and no yield strain was observed for mucoid Alg.

**Fig. 5:**
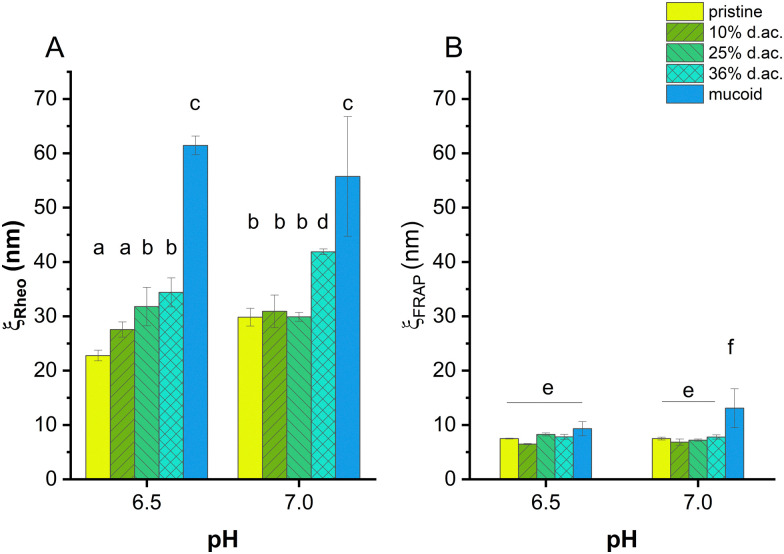
Mesh size of alginate hydrogels obtained by rheological measurements (*ξ*_Rheo_, A) and FRAP (*ξ*_FRAP_, B). The generalized Maxwell model was applied to calculate the mesh size. The mesh size was dependent on the d. ac. at pH 6.5, while no trend, except 36% d. ac., was observed at pH 7. Mucoid Alg showed the highest mesh size under both conditions. Modelling the mesh size using FRAP showed no influence of the acetylation on the mesh size. While the gels composed of mucoid Alg showed statistically significant higher mesh sizes at pH 7, no statistically significant difference was observed at pH 6.5.

Rheological measurements were performed on hydrogels to determine how the acetylation might influence the gels’ viscoelastic properties and stability. In amplitude sweeps, it was observed that the acetylation of Alg led to an increase in the linear viscoelastic region (LVER, Fig. 4(A) and (B)) with a steady decrease in storage modulus with increasing d. ac. at both investigated pH. The softening effect was less pronounced at pH 7 compared to pH 6.5 as *G*′ dropped from 319 Pa to 103 Pa and 647 Pa to 191 Pa, respectively, which mirrors the results from the compression tests described above. The increase in elastic properties is further confirmed by the decreasing loss factor (tan *δ*, Table 1), which describes the ratio of lost energy due to dissipation (viscous properties) and stored energy (elastic properties). The LVER of the hydrogel, as indicated by our results, is directly related to the d. ac. and is increasing with increasing d. ac., with 25% and 36% d. ac. up to 20% of deformation. The increased LVER could be caused by a decreased crosslinking density, as indicated by the mesh size, *ξ*_rheo_ (Fig. 5(A)), obtained from the frequency sweeps (Fig. 4(C) and (E)). Wloka *et al.* performed rheological measurements on native PA biofilms produced from the strains FDR1 and FDR1153, the latter incapable of Alg acetylation.^71^ The LVER of FDR1 and FDR1153 were tested to be 10% and 1%, respectively, for a Ca^2+^ undersaturated condition,^71^ which is in agreement with our values at both investigated pHs.

**Table 1:** Results for the equilibrium shear modulus *G*_∞_, used in the generalized Maxwell model on the data obtained from the frequency sweeps. The loss factor presented was taken at 0.1% strain, well within the LVER. *G*_∞_ and loss factor (tan *δ*) decrease with increasing d. ac. The reduced *G*_∞_ indicates that the gels are softer, but the tanδ decreases, indicating that less energy is dissipated

Degree of acetylation	pH 7		pH 6.5	
	*G*_∞_ (Pa)	Loss factor	*G*_∞_ (Pa)	Loss factor
Pristine	319 ± 15	0.12 ± 0.01	647 ± 71	0.25 ± 0.05
10%	209 ± 20	0.07 ± 0.01	366 ± 43	0.14 ± 0.01
25%	284 ± 18	0.05 ± 0.01	247 ± 59	0.05 ± 0.01
36%	103 ± 3	0.05 ± 0.01	191 ± 35	0.06 ± 0.01
Mucoid	50 ± 32	0.07 ± 0.01	33 ± 2	0.06 ± 0.01

Additionally, the yield strain was increased from 4% to 54% for pristine and 36% d. ac., respectively, indicating an increased resistance towards irreversible deformation by flow (Fig. 4(E)), with a maximum of 94% at 25% d. ac. Hydrogels composed of mucoid Alg did not show any yield strain in the investigated strain regime. It was reported by Wloka *et al.* that native 24 h-old biofilms have a yield strain higher than 200%.^71^ Conditioning the gels in a slightly acidic medium increased the storage modulus, decreased the LVER (Fig. 4(B)), and reduced the yield strain (Fig. 4(E)). The conditioning could have caused unreacted CaCO_3_ to decompose and release Ca^2+^ ions, leading to increased crosslinking and, hence, increased *G*_∞_ (Table 1) and *G*′. The decrease of the LVER could be caused by the higher saturation of G-units crosslinked by Ca^2+^, which are more rigid, hence higher *G*′, but are less elastic and break earlier under strain or hydration during incubation in buffered solution. Hydration could lead to pretension, which causes the gel to transition to a liquid state at lower strains. The loss factor increased with increasing storage modulus in the hydrogels composed of pristine and 10% d. ac. Swelling could cause the network to expand and get more rigid. This could explain a higher *G*′ and, due to increased hydration, increased energy dissipated from the network in the surrounding solution.

Previous studies comparing Alg-based formulations with CF sputum have shown that 2–3 mg ml^−1^ Alg closely matched the rheological properties of CF sputum.^39^ Due to the strong influence of the acetylation on the rheological properties, underestimation of the Alg concentration could occur within the models, impacting the predictions of drug diffusion in the models. Using a lower Alg concentration might lead to a too loose network, through which the antibiotic diffuses easier than through the native biofilm. As the Alg concentration directly influences the network's density and regulates the capacity of cation exchange through the M blocks, the Alg concentration should not deviate too far from the native case. Interactive filtering *via* charge–charge interaction or H-bridges is also underestimated as this is directly influenced by the Alg concentration.

A rheological mesh size *ξ*_rheo_ was modelled based on the frequency-dependent response of the gel to stress.^59,60,72^ It was observed that *ξ*_rheo_ was increasing with increasing d. ac., ranging from 30 nm (for pristine and 10% d. ac.) to 40 nm (36% d. ac., Fig. 5(A)). Furthermore, *ξ*_rheo_ obtained through the modelling decreased according to the increased *G*′ for all samples at pH 6.5 compared to pH 7.0. Excessive swelling could be excluded as this would lead to increased mesh size within the hydrogel.

Through FRAP experiments, it was also possible to model a mesh size, *ξ*_FRAP_, through which solutes, *e.g.*, proteins, antibiotics, or nutrients, can diffuse through the gel. Using FITC-dextrans of different molecular weights, it was possible to determine at which size the dextran reptation occurs.^73^ Reptation, an elongation along one axis and, hence, deviation from spherical shape, was observed in two different ways: as a theoretically negative mesh size due to increased diffusion coefficient or as a mesh size smaller than the hydrodynamic radius of the dextran. Therefore, it was possible to determine an effective pore radius of 7–9 nm at pH 7 for the non-acetylated and acetylated Alg, and 11 nm for mucoid Alg, as shown in Fig. 5(B).

The commercially available Alg, representing non-acetylated Algs, has a lower M/G ratio than mucoid Alg. The larger poly-M blocks have reduced affinity for Ca^2+^ ions. They are moving freely and interacting with solutes diffusing through the gel.^70^ The FRAP experiments have shown that *ξ*_FRAP_ of Alg hydrogels first decreased until 10% d. ac. before increasing with higher d. ac. *ξ*_FRAP_ decreased with decreasing pH for pristine and 10% d. ac. However, *ξ*_FRAP_ increased with decreasing pH for d. ac. 25% and 36%. Mucoid Alg showed a drastic reduction in pore size from 13 nm at pH 7 to 9.5 nm at pH 6.5, indicating that the individual Alg chains interact more with each other. While *ξ*_FRAP_ of mucoid Alg were significantly higher than the chemically acetylated Alg, despite its higher molecular weight at pH 7, no statistically significant difference was found between the different samplesat pH 6.5. It was also found that the *ξ*_rheo_ was twice higher than *ξ*_FRAP_. There are several possible reasons why *ξ*_rheo_ is consistently larger than *ξ*_FRAP_. Physical entanglements are not included in the model to calculate *ξ*_rheo_, but they could greatly reduce the effective pore size experienced by diffusing macromolecules. Further, entanglement increases with molecular weight and could lead to larger effective differences with higher molecular weight, as observed for the mucoid Alg. M-blocks of the Alg chain could cause such entanglements. Also, we do not know the pattern of M-blocks, G-blocks, and acetylation. The rheological mesh size reflects the average size of all crosslinking points. High polydispersity between crosslinking points will obscure the existence of regions with tighter crosslinking. However, *ξ*_FRAP_ is mainly sensitive to the existence of regions of such smaller pore sizes. Transient weak binding of M-blocks could also slow diffusion while providing negligible influence on *ξ*_rheo_.

### Interaction of Alg with tobramycin

Tobramycin, commonly used to combat PA in cystic fibrosis patients, is an aminoglycoside with a pH-dependent charge, showing complete protonation below pH 7.^74^ It can be assumed that tobramycin is fully protonated at the active site as the pH of CF biofilms is in the range of 5 to 7.^13^ Tobramycin has been proven to interact strongly with PA biofilms, especially with Alg.^15^ Its strong double-layer interactions with Alg result in drastic changes in the volume of Alg-based hydrogels.^14,22^ M-rich Alg was shown to bind more aminoglycosides than G-rich Alg, resulting in a pronounced shrinkage upon antibiotic incubation.^22^ Bacterial Alg is known to exhibit higher diffusion retardation of aminoglycosides than seaweed Alg, improving bacterial survival.^14–16^

**Fig. 6:**
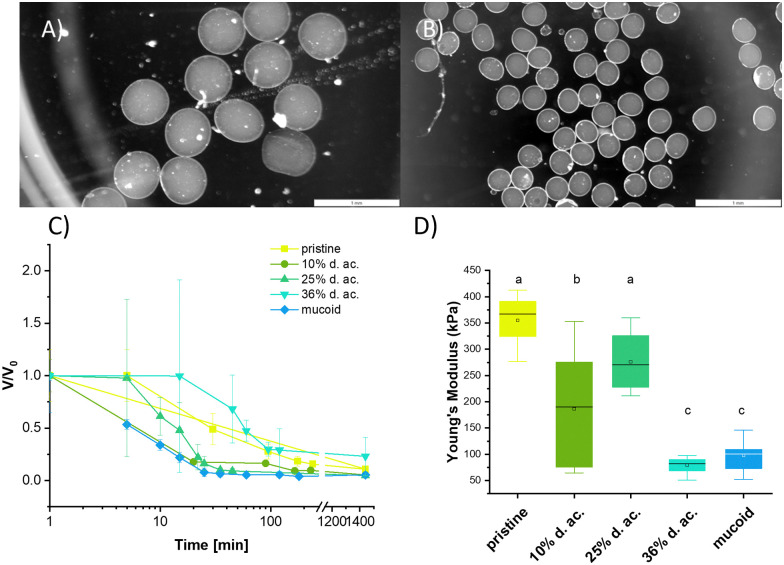
Microbeads (A) before and (B) after 24 h of incubation with tobramycin. (C) Time-resolved shrinkage of Alg microbeads for different Alg compositions. (D) The Young's moduli of microbeads made from different Alg after tobramycin incubation (0.699 mg ml^−1^) for 24 h at pH 6.5. Scale bar 1 mm.

We used Alg microbeads produced by inkjet as 3D models to investigate the time-dependent shrinkage upon incubation in a 0.7 mg ml^−1^ tobramycin solution at pH 6.5 and perform compression tests to investigate the change in Young's modulus. Examples of the microbeads before and after tobramycin incubation are shown in Fig. 6(A) and (B). The radii before and after the incubation can be seen in Table 2. After printing, all Alg derived from pristine Alg had similar radii with no observable trend. Upon incubation with tobramycin, all beads shrink drastically due to ion exchange and uptake of tobramycin^5+^ ions into the network. After 24 h of incubation, 10% and 25% d. ac. Alg microbeads were significantly smaller, whereas microbeads from 36% d. ac. Alg were significantly larger than pristine Alg beads. Microbeads made from mucoid Alg were initially significantly larger than the other Algs but in similar size ranges after incubation in tobramycin solution. Furthermore, the time-resolved shrinkage analysis showed variations in the shrinkage rate of tobramycin (Fig. 6(C)). It is instructive to correlate the shrinkage and rate of shrinkage with the amount of tobramycin found in a tobramycin-saturated gel (Table 2). This comparison indicates that low to moderate d. ac. bind high amounts of tobramycin in the hydrogel, whereas higher d. ac. reduces its uptake. The reduction of tobramycin uptake at high d. ac. could be caused by either increased lipophilicity on the acetylated M regions of the Alg chains or decreased charge density on the Alg backbone.^15,16,20,34^

**Table 2:** Comparison of starting radii of Alg microbeads with different degrees of acetylation before and after 24 h of incubation in tobramycin and the tobramycin mass relative to the Alg mass in gels after 24 h incubation

Sample	*r*_0_ (μm)	*r*_24 h_ (μm)	Tobramycin (μg_Tobramycin_ mg^−1^_gel_)
Pristine	208 ± 18	100 ± 5	34 ± 5
10% d. ac.	185 ± 10	69.0 ± 9	46 ± 6
25% d. ac.	199 ± 2	72.0 ± 3	60 ± 13
36% d. ac.	198 ± 2	112 ± 17	31 ± 3
Mucoid	305 ± 8	103 ± 5	46 ± 2

Compression tests performed on the incubated Alg beads showed that the Young's modulus of the hydrogel drastically increased (up to 40-fold for 25% d. ac., Fig. 6(D)). The lower increase in Young's Modulus for 36% d. ac. correlates with the lower amount of tobramycin integrated into the Alg network. As the material is compressed to a much smaller volume, the increase in Young's modulus is expected, but the strong contrast between mucoid, pristine and acetylated Alg was surprising. Although pristine, mucoid and 36% d. ac. showed similar size after incubation in tobramycin solution, the Young's modulus of pristine Alg was significantly higher compared to either mucoid and 36% d. ac. The lower Young's modulus for 36% d. ac. and mucoid Alg could be caused by reduced affinity of the Alg backbone to the drug. Although similar tobramycin contents per mg of Alg gel were found between 10% d. ac. and mucoid samples, 36% d. ac. showed the highest similarities in the stiffness of the microbeads after incubation. The reduced diffusion rate of tobramycin and its lower total loading of tobramycin for 36% d. ac. compared to the increase of diffusion rate and total loading with d. ac. up to 25% d. ac. is striking but remains unexplained. Surprisingly, mucoid Alg showed the fastest decrease in size. After 24 h of incubation, mucoid Alg beads showed the smallest volume fractions compared to the original size, indicating that the beads were hydrated higher than the seaweed-derived Alg (acetylated and pristine). After complete saturation of all binding partners, the material was compacted to a similar size as the other samples.

Tobramycin-incubated microbeads exposed to 70 kDa FITC-Dextran (*R*_h_ = 5.8 nm^44^) and rhodamine B (*R*_h_ = 0.78 nm^75^) solutions did not take up the dyes inside the hydrogels. The contraction indicates that tobramycin causes a significant increase in crosslinking density due to the exchange of a divalent cation (Ca^2+^) to a five-times positively charged species (tobramycin^5+^). The contraction and the higher charge density in the hydrogel can contribute to the negligible diffusion of rhodamine B (positively charged) and FITC-dextran (negatively charged). Although the final sizes are statistically not significantly different between pristine, 36% d. ac. and mucoid, the Young's modulus was drastically different.

**Fig. 7:**
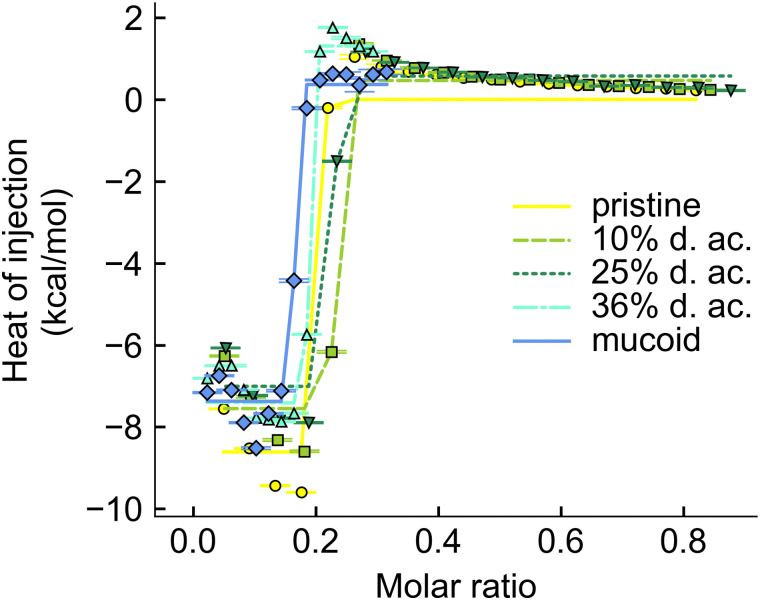
Integrated thermogram obtained from titrating tobramycin into Alg solution during the ITC experiments.

The interaction between Alg and tobramycin was also investigated using ITC. This method is often used in protein–ligand^46^ or polyelectrolyte–polyelectrolyte,^46,76^ or drug-ligand^76^ studies to investigate the thermodynamic parameters (Δ*H*, Δ*S*, Δ*G*, and *K*_D_) of a reaction. The dissociation constant, *K*_D_, describes the equilibrium of the reaction and indicates how strong the binding reaction is. The binding between tobramycin and all different Alg was found to be so strong that the *K*_D_ was smaller than 10^−12^ M (Fig. 7). The binding reaction is highly enthalpic but decreases with increasing degree of acetylation. The enthalpic reaction seems mainly driven by charge interactions, given by the molar binding ratio of ∼0.2, which corresponds to the charge ratio between the monomeric units of Alg (one negative charge per unit) and the charges on the fully protonated tobramycin^5+^ ion, as well as the abrupt saturation of all binding sites. The increase in the heat of injection at the beginning of the experiments could be interpreted as increased reactivity of the binding partners. This decrease in reaction enthalpy indicates a deviation from an ideal 1 : 1 reaction between the reactive groups, however, a detailed investigation into the binding reaction is out of the scope of this paper. Besides the reduction of the reaction enthalpy, acetylation of Alg did not alter the binding reaction. The binding reaction between Alg and tobramycin^5+^ is fast, strong, and independent of the acetyl side group. The slight offsets from the ideal molar ratio of 0.2 can be explained by uncertainties during weighing in, dilution errors and deviations in the stock solutions after dialysis.

## Conclusions

In their native biofilm, mucoid PA produces Alg as the predominant component of their EPS. The secreted Alg is, unlike seaweed Alg, rich in M-units, which are acetylated. We have developed a robust protocol to target specific d. ac. to match PA Alg to study the influence of acetylation on biofilm properties. We showed that the acetylation of the Alg M-units mainly impacts the mechanical properties by drastically decreasing the Young's and storage moduli while increasing the linear viscoelastic region. The pH-dependent mechanical properties indicate that acetylated Alg is completely different from unmodified seaweed-derived Alg. We also proved that M-rich, synthetically acetylated Alg more closely matches native bacterial Alg regarding mechanical properties and antibiotic binding and retention. It is still unclear why PA is modifying the Alg it produces. However, from our comparisons, it is reasonable to assume that it affords increased environmental protection while still providing large pores for the diffusion of other compounds through the biofilm. It also serves to reduce the pH sensitivity of the Alg-gels’ mechanical properties, which is beneficial as PA regulates its acidic environment. Acetylation seemed only to influence the binding interactions with tobramycin marginally. Still, it affected the shrinking of the Alg hydrogel, increasing it at low acetylation and reducing it at high acetylation. The influence of acetylation on the mechanical and structural properties of Alg suggests that it can greatly impact the physical and biological functions of PA biofilms. Our synthetic platform captures these aspects and allows us to build a platform for further work testing drug delivery systems, (bio)molecular interactions, and mechanical properties.

## Author contributions

Stephan Schandl: writing – original draft, visualization, methodology, formal analysis, investigation. Goodness Osondu-Chuka: writing – review and editing, visualization, methodology, formal analysis, investigation. Giuseppe Guagliano: writing – review and editing methodology, formal analysis, investigation, validation. Stjepan Perak: investigation, methodology. Paola Petrini: writing – review & editing, supervision, formal analysis. Francesco Briatico Vangosa: writing – review & editing, supervision, formal analysis. Erik Reimhult: writing – review & editing, supervision, conceptualization, formal analysis, funding acquisition, project administration, resources. Olivier Guillaume: writing – review & editing, supervision, conceptualization, formal analysis, funding acquisition, project administration, resources.

## Data availability

The data supporting this article have been included as part of the ESI.† Data for this article are available at researchdata.tuwien.at https://doi.org/0.48436/0k31k-42d13.

## Conflicts of interest

There are no conflicts to declare.

## Supplementary Material

TB-013-D4TB02675F-s001

## References

[cit1] Hall-Stoodley L., Costerton J. W., Stoodley P. (2004). Nat. Rev. Microbiol..

[cit2] Weber S. D., Ludwig W., Schleifer K.-H., Fried J. (2007). Appl. Environ. Microbiol..

[cit3] Flemming H.-C. (1993). Water Sci. Technol..

[cit4] Gaylarde C. C., Morton L. H. G. (1999). Biofouling.

[cit5] Gaylarde C. C., Gaylarde P. M. (2005). Int. Biodeterior. Biodegrad..

[cit6] Hallmann C., Stannek L., Fritzlar D., Hause-Reitner D., Friedl T., Hoppert M. (2013). FEMS Microbiol. Ecol..

[cit7] Ronin D. , Felix R. B., Williams C. M., Mannuel S. A., Goeres D., Summers J., LaFleur J. E. and Kjellerup B. V., in Antibiofilm Strategies: Current and Future Applications to Prevent, Control and Eradicate Biofilms, ed. K. Richter and K. N. Kragh, Springer International Publishing, Cham, 2022, pp. 61–97 10.1007/978-3-031-10992-8_4

[cit8] Khabipova N., Valeeva L., Shaidullina E., Kabanov D., Vorobev V., Gimadeev Z., Sharipova M. (2023). Bionanoscience.

[cit9] Trautner B. W., Darouiche R. O. (2004). Am. J. Infect. Control.

[cit10] Høiby N., Ciofu O., Bjarnsholt T. (2010). Future Microbiol..

[cit11] Hassett D. J., Korfhagen T. R., Irvin R. T., Schurr M. J., Sauer K., Lau G. W., Sutton M. D., Yu H., Hoiby N. (2010). Expert Opin. Ther. Targets.

[cit12] Ghafoor A., Hay I. D., Rehm B. H. A. (2011). Appl. Environ. Microbiol..

[cit13] Guillaume O., Butnarasu C., Visentin S., Reimhult E. (2022). Biofilm.

[cit14] Cao B., Christophersen L., Kolpen M., Jensen P. Ø., Sneppen K., Høiby N., Moser C., Sams T. (2016). PLoS One.

[cit15] Nichols W. W., Dorrington S. M., Slack M. P., Walmsley H. L. (1988). Antimicrob. Agents Chemother..

[cit16] Tseng B. S., Zhang W., Harrison J. J., Quach T. P., Song J. L., Penterman J., Singh P. K., Chopp D. L., Packman A. I., Parsek M. R. (2013). Environ. Microbiol..

[cit17] Skjåk-Bræk G., Grasdalen H., Larsen B. (1986). Carbohydr. Res..

[cit18] Schweiger R. G. (1962). J. Org. Chem..

[cit19] Schweiger R. G. (1962). J. Org. Chem..

[cit20] Skjåk-Bræk G., Zanetti F., Paoletti S. (1989). Carbohydr. Res..

[cit21] Butnarasu C., Caron G., Pacheco D. P., Petrini P., Visentin S. (2022). Mol. Pharmaceutics.

[cit22] Heriot M., Nottelet B., Garric X., D’Este M., Richards G. R., Moriarty F. T., Eglin D., Guillaume O. (2019). Int. J. Biol. Macromol..

[cit23] Singh N., Romero M., Travanut A., Monteiro P. F., Jordana-Lluch E., Hardie K. R., Williams P., Alexander M. R., Alexander C. (2019). Biomater. Sci..

[cit24] Xu Y., Liu Q., Wang B., Li Q., Chen Y., Yang Y., Zhu Z., Gong D., Zhang C., Wang G., Qian H. (2024). Biomater. Sci..

[cit25] Iglesias Y. D., Bambeke F. V. (2020). Antimicrob. Agents Chemother..

[cit26] Sarkisova S., Patrauchan M. A., Berglund D., Nivens D. E., Franklin M. J. (2005). J. Bacteriol..

[cit27] Smith D. J., Anderson G. J., Bell S. C., Reid D. W. (2014). J. Cystic Fibrosis.

[cit28] Jacobs H. M., O’Neal L., Lopatto E., Wozniak D. J., Bjarnsholt T., Parsek M. R. (2022). J. Bacteriol..

[cit29] Ramos P. E., Silva P., Alario M. M., Pastrana L. M., Teixeira J. A., Cerqueira M. A., Vicente A. A. (2018). Food Hydrocolloids.

[cit30] Quong D., Neufeld R. J., Skjåk-Bræk G., Poncelet D. (1998). Biotechnol. Bioeng..

[cit31] Ng W. L., Shkolnikov V. (2024). Bio-Des. Manuf..

[cit32] Hunt N. C., Hallam D., Karimi A., Mellough C. B., Chen J., Steel D. H. W., Lako M. (2017). Acta Biomater..

[cit33] Ning E., Turnbull G., Clarke J., Picard F., Riches P., Vendrell M., Graham D., Wark A. W., Faulds K., Shu W. (2019). Biofabrication.

[cit34] SkjÅk-Bræk G., Paoletti S., Gianferrara T. (1989). Carbohydr. Res..

[cit35] Chanasit W., Gonzaga Z. J. C., Rehm B. H. A. (2020). Appl. Microbiol. Biotechnol..

[cit36] Limoli D. H., Whitfield G. B., Kitao T., Ivey M. L., Davis M. R., Grahl N., Hogan D. A., Rahme L. G., Howell P. L., O’Toole G. A., Goldberg J. B. (2017). mBio.

[cit37] Schindelin J., Arganda-Carreras I., Frise E., Kaynig V., Longair M., Pietzsch T., Preibisch S., Rueden C., Saalfeld S., Schmid B., Tinevez J.-Y., White D. J., Hartenstein V., Eliceiri K., Tomancak P., Cardona A. (2012). Nat. Methods.

[cit38] Gubernator J., Drulis-Kawa Z., Kozubek A. (2006). Int. J. Pharm..

[cit39] Pacheco D. P., Butnarasu C. S., Briatico Vangosa F., Pastorino L., Visai L., Visentin S., Petrini P. (2019). J. Mater. Chem. B.

[cit40] Guagliano G., Volpini C., Camilletti J., Donnaloja F., Briatico-Vangosa F., Visai L., Petrini P. (2023). Biofabrication.

[cit41] Wilson J. L., Najia M. A., Saeed R., McDevitt T. C. (2014). Biotechnol. Bioeng..

[cit42] Moresi M., Bruno M. (2007). J. Food Eng..

[cit43] Richbourg N. R., Peppas N. A. (2021). Macromolecules.

[cit44] Wallace M., Adams D. J., Iggo J. A. (2013). Soft Matter.

[cit45] Langevin D., Rondelez F. (1978). Polymer.

[cit46] Scheuermann T. H., Brautigam C. A. (2015). Methods.

[cit47] Zhao H., Piszczek G., Schuck P. (2015). Methods.

[cit48] Brautigam C. A. , in Methods in Enzymology, ed. J. L. Cole, Academic Press, 2015, vol. 562, pp. 109–133 10.1016/bs.mie.2015.05.001 26412649

[cit49] Pawar S. N., Edgar K. J. (2012). Biomaterials.

[cit50] Straatmann A., Borchard W. (2003). Eur. Biophys. J..

[cit51] Kohn R. (1975). Pure Appl. Chem..

[cit52] Pawar S. N., Edgar K. J. (2011). Biomacromolecules.

[cit53] Evans L. R., Linker A. (1973). J. Bacteriol..

[cit54] Straatmann A. , Windhues T. and Borchard W.,Effects of acetylation on thermodynamic properties of seaweed alginate in sodium chloride solutions, Berlin, Heidelberg, 2004

[cit55] Degrassi A., Toffanin R., Paoletti S., Hall L. D. (1998). Carbohydr. Res..

[cit56] Schürks N., Wingender J., Flemming H. C., Mayer C. (2002). Int. J. Biol. Macromol..

[cit57] Agasty A., Wisniewska A., Kalwarczyk T., Koynov K., Holyst R. (2021). ACS Appl. Polym. Mater..

[cit58] Gao Q., Kim B.-S., Gao G. (2021). Mar. Drugs.

[cit59] Turco G., Donati I., Grassi M., Marchioli G., Lapasin R., Paoletti S. (2011). Biomacromolecules.

[cit60] Sardelli L., Tunesi M., Briatico-Vangosa F., Petrini P. (2021). Soft Matter.

[cit61] Machado A. H. E., Lundberg D., Ribeiro A. J., Veiga F. J., Miguel M. G., Lindman B., Olsson U. (2013). Langmuir.

[cit62] Wilton M., Charron-Mazenod L., Moore R., Lewenza S. (2016). Antimicrob. Agents Chemother..

[cit63] Massip-Copiz M. M., Santa-Coloma T. A. (2018). Eur. J. Cell Biol..

[cit64] Yang J., Chen S., Fang Y. (2009). Carbohydr. Polym..

[cit65] Cao Y., Shen X., Chen Y., Guo J., Chen Q., Jiang X. (2005). Biomacromolecules.

[cit66] Chan E.-S., Lee B.-B., Ravindra P., Poncelet D. (2009). J. Colloid Interface Sci..

[cit67] Ribeiro A. C. F., Sobral A. J. F. N., Simões S. M. N., Barros M. C. F., Lobo V. M. M., Cabral A. M. T. D. P. V., Veiga F. J. B., Santos C. I. A. V., Esteso M. A. (2011). Food Chem..

[cit68] Lee J. W., Ashby R. D., Day D. F. (1996). Carbohydr. Polym..

[cit69] Hu C., Lu W., Mata A., Nishinari K., Fang Y. (2021). Int. J. Biol. Macromol..

[cit70] Kohn R. (1975). Pure Appl. Chem..

[cit71] Wloka M., Rehage H., Flemming H. C., Wingender J. (2005). Biofilms.

[cit72] Karvinen J., Ihalainen T. O., Calejo M. T., Jönkkäri I., Kellomäki M. (2019). Mater. Sci. Eng., C.

[cit73] de Gennes P. G. (2003). J. Chem. Phys..

[cit74] Alkhzem A. H., Woodman T. J., Blagbrough I. S. (2020). ACS Omega.

[cit75] Rani S. A., Pitts B., Stewart P. S. (2005). Antimicrob. Agents Chemother..

[cit76] Powell L. C., Pritchard M. F., Ferguson E. L., Powell K. A., Patel S. U., Rye P. D., Sakellakou S.-M., Buurma N. J., Brilliant C. D., Copping J. M., Menzies G. E., Lewis P. D., Hill K. E., Thomas D. W. (2018). npj Biofilms Microbiomes.

