# Supplementary material for: Acetylation of alginate enables the production of inks that mimic the chemical properties of P. aeruginosa biofilm
Source: J Mater Chem B. 2025 Jan 22;13(8):2796–809. doi: 10.1039/d4tb02675f (PMC11773326; doi:10.1039/d4tb02675f)
Supplement: TB-013-D4TB02675F-s001 [file TB-013-D4TB02675F-s001.pdf]

Supplementary Information (SI) for Journal of Materials Chemistry B.
This journal is © The Royal Society of Chemistry 2025

Supporting Information

Acetylation of alginate enables the production of inks that mimic the chemical properties of *P. aeruginosa* biofilm

Stephan Schandl[a,b,*], *Goodness Osondu-Chuka*[c], *Giuseppe Guagliano*[d], *Stjepan Perak*[a], *Paola Petrini*[d], *Francesco Briatico Vangosa*[d], *Erik Reimhult*[c], *Olivier Guillaume*[a,b]

[a] Institute of Materials Science and Technology, Technische Universität Wien, Vienna, Austria

[b] Austrian Cluster of Tissue Regeneration (https://www.tissue-regeneration.at)

[c] Department of Bionanosciences, Institute of Colloid and Biointerface Science, BOKU University

[d] Department of Chemistry, Materials and Chemical Engineering 'G Natta', Politecnico di Milano, Milan, Italy

[*]corresponding author

Email: stephan.schandl@tuwien.ac.at

Table of content

Alginate Acetylation 2

Characterization of Acetylated Alginate Inks and Hydrogels 4

References 7

Alignate Acetylation

The acetylation of alginate was performed according to modified literature.[1] The modification of the protocol was achieved by small and easy to apply changes in the protocol. Pyridine and acetic anhydride (Ac_2O) were dried over 4 Å molecular sieve. Different concentrations (2, 3.5, 5.5 and 12 vol%) of Ac_2O were tested in the reactions. After complete crosslinking, the alginate beads were filtered off, pre-dried/washed with 50 ml pyridine before drying in pyridine over-night in N_2-atmosphere. The beads were filtered off and incubated in a mixture of pyridine and Ac_2O (100 ml in total) with corresponding vol% of Ac_2O and beads stirred for 24 h at either 38 °C or room temperature (RT). The beads were filtered off, washed first twice with acetone and twice with water (100 ml solvent and 5 min of stirring per washing step). The beads were filtered off and dissolved in 50 ml 0.2 M EDTA before dialysis in 0.9 wt% NaCl. The solvent was exchanged after every 3-4 h for 2 days after which DI was used as solvent. After 48 h dialysis against DI, the solution was concentrated to roughly half the volume using a rotary evaporator and lyophilized. The degree of acetylation (d. ac.) was determined using 1H-NMR after enzymatic digestion. The d. ac. was calculated according to literature,[1] using the following formula:

$$d.A. = \frac{\frac{I_{Ac}}{3}}{\frac{I_{BB}}{5}} * 100\%$$

With d. ac., IAc and IBB being the degree of Acetylation, the integral of the acetyl peak and the integral of the alginate backbone (up and downfield of the solvent peak), respectively. The factors 3 and 5 are derived from the three proton per acetyl side group and five protons per monomeric unit. The ^{1}H-NMR of all tested Ac2O concentrations is depicted in Figure S1.

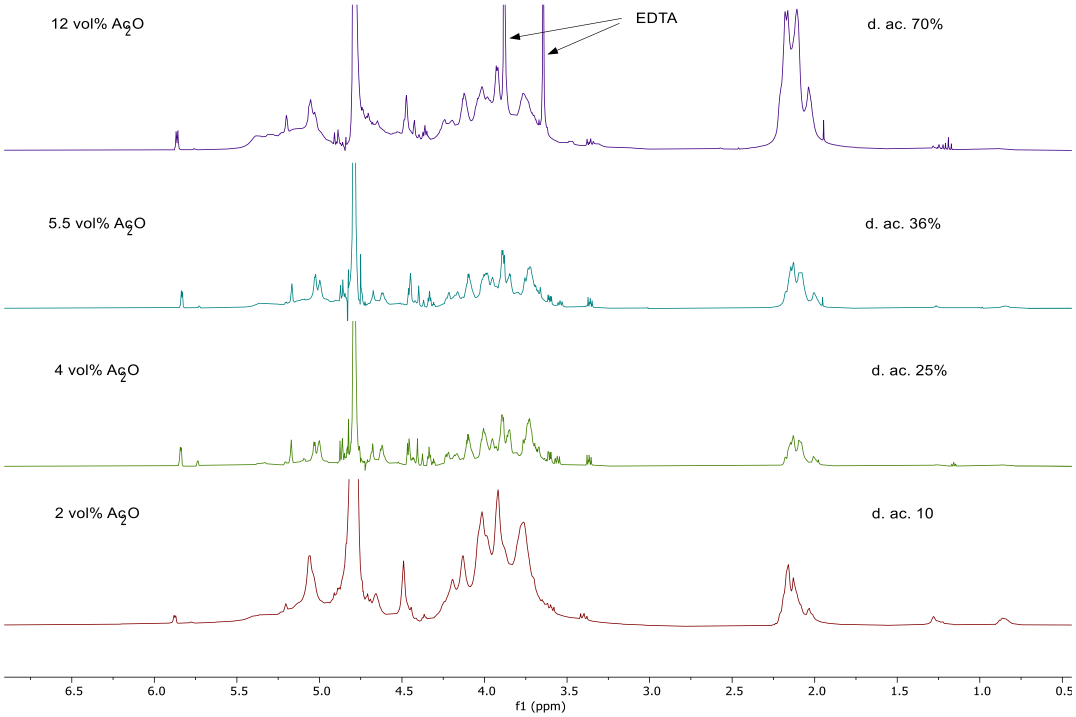

Figure S1: 1H-NMR of all modified alginate using different concentrations of acetic anhydride. Increasing Ac_2O in the reaction solution has led to an increase in degree of acetylation. The high singlet signals in the sample with 12 vol% Ac_2O at 3.88 and 3.64 ppm are derived from leftover EDTA.

All alginates up to a d. ac. of 36% were completely soluble without limitations. At an d. ac. of 70% solubility was drastically reduced and the solution always appeared inhomogeneous. The efficiency of the enzymatic digestion was reduced with increasing d. ac.. At d. ac. of 70% almost no degradation can be assumed. With the alginate of PA having a d. ac. of ~ 34%, a concentration 5.5 vol% (100 ml per gram of alginate powder) was decided to be optimal. The reaction was then performed at 38 °C, which is used in literature[1, 2] and at room temperature to investigate the necessity of slightly elevated temperature. The results from the temperature comparison can be seen in the table below.

Table S 1 Comparison of the reaction temperatures of RT and 38 °C

Ac_2O concentration	Temperature (°C)	d. ac. (%)
2 vol%	RT	15.3
	38	15.8
4 vol%	RT	25.2
	38	25.3
5.5 vol%	RT	35.9
	38	36
12 vol%	RT	71
	38	73

The reaction temperature did not influence the results of the reaction. Although small deviations are observable, they can also be derived from inaccuracies in the ^1H-NMR analysis. It can be concluded that slightly increased temperatures are not necessary to modify low degrees of acetylation at a constant time of 24 h.

Characterization of Acetylated Alginate Inks and Hydrogels

Inkjet printing of alginate inks was performed at various pressures at set open and cycle times of 1 and 180 ms, respectively. The minimum pressure at which the formulation could be jetted was 35 kPa. The pressure was increased in 5 kPa increments. Incomplete jetting of individual droplets was observed at low pressure (Figure S2), while higher pressure (45 – 60 kPa) ensured the production of beads with narrow size distribution. In both cases, the pinch-off is visible as an artefact of the inkjet printing method.

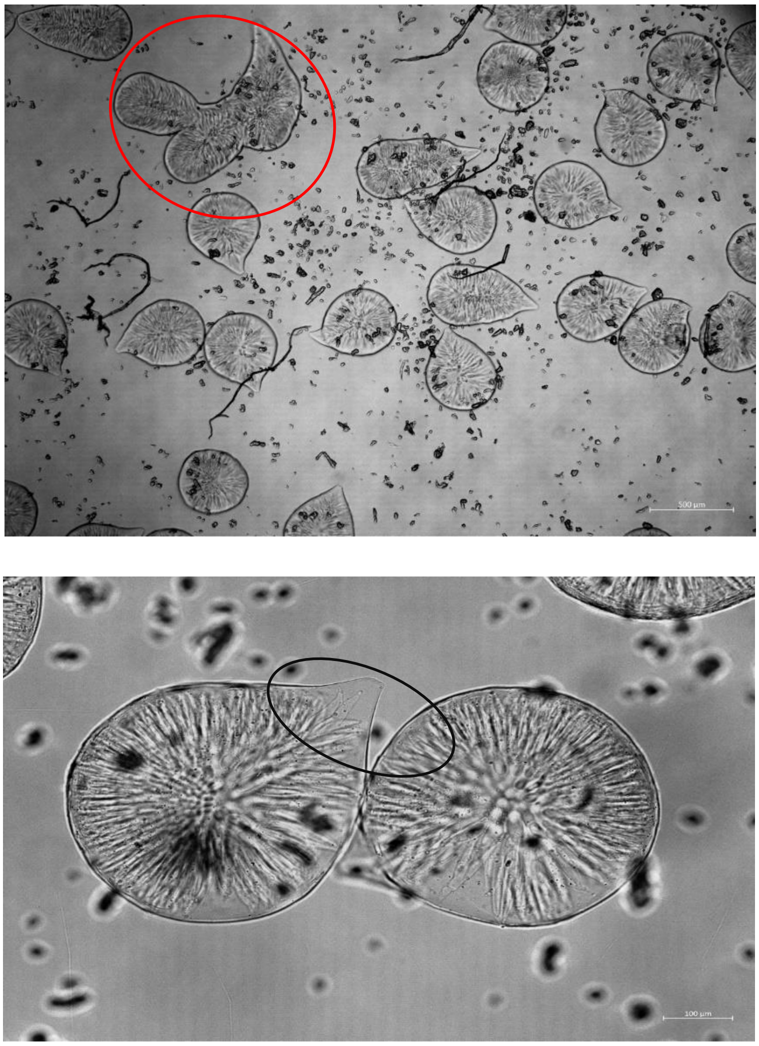

Figure S2: Microbeads produced at 35 kPa. Top: General overview of the printed beads. Due to low-pressure jetting, ink may accumulate on the nozzle, leading to multiple dorplets merging before detaching from the inkjet head. (red circle, scalebar: 500 µm). Bottom: Higher magnification on the microbeads. The pinch-off, typical of inkjet, is visible on the beads (black circle, scalebar 100 µm).

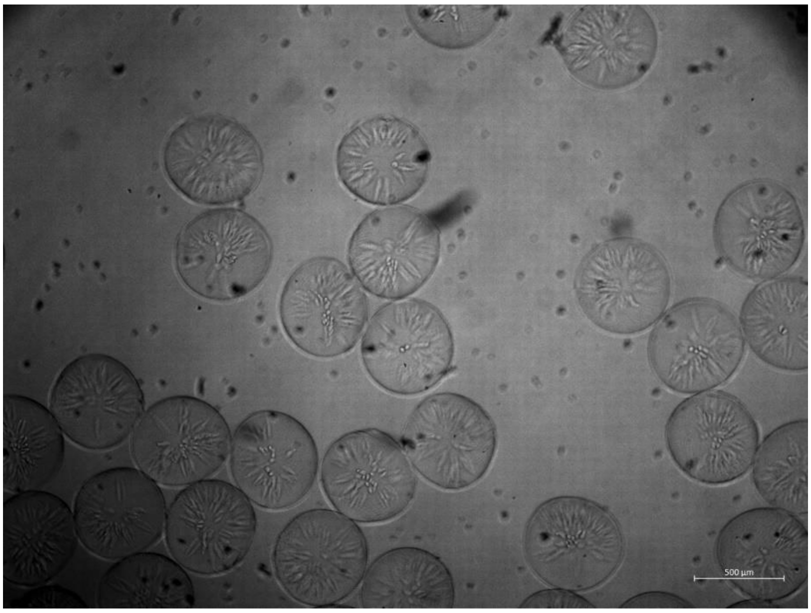

Figure S3: At 55 kPa pressure, the printing was highly reproducible, and homogeneously sized beads were obtained (scale bar 500 µm).

However, at pressures higher than 60 kPa, the beads were too soft, and when they hit the crosslinking bath, they could not withstand the forces and were deformed (Figure S4).

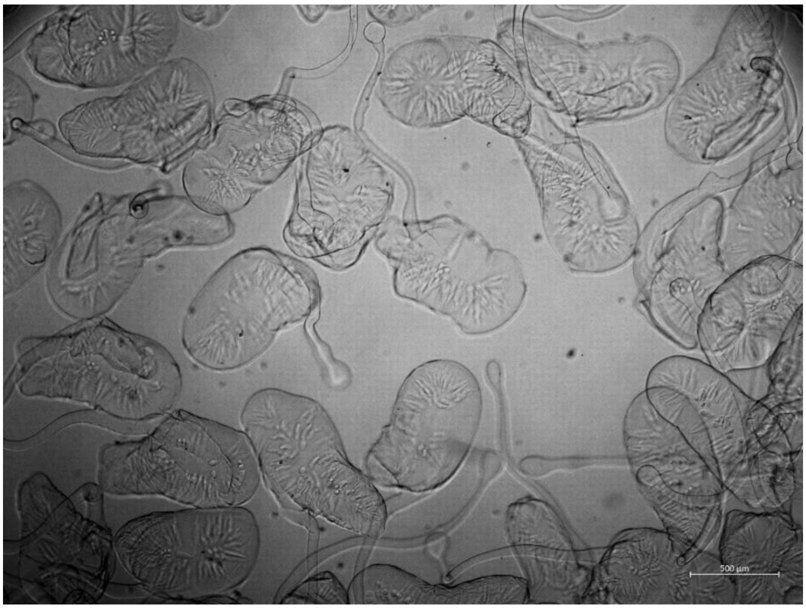

Figure S4: Microparticles printed at 80 kPa pressure. The droplets deformed when they came into contact with the liquid surface before their shape could be stabilized by Ca^{2+} crosslinking (scale bar 500 µm).

The density (Figure S5) of the used alginate inks were not statistically significant different and were between the tested groups.

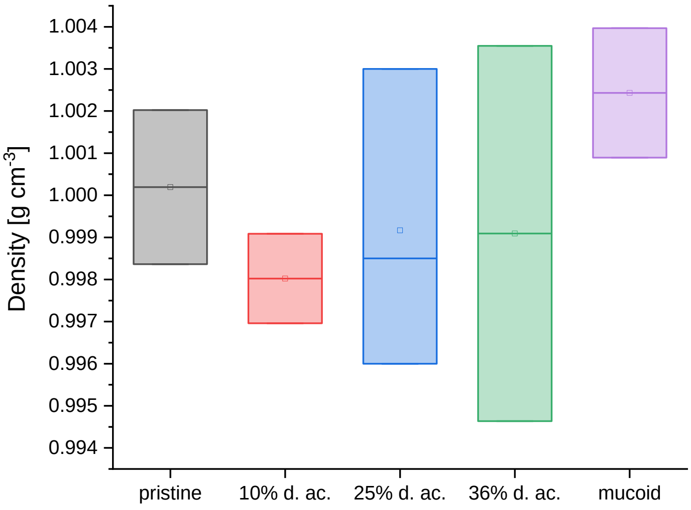

Figure S5: Measured densities of 0.5 wt% alginate solutions.

The surface tension of alginate is depicted in Figure S6 and showed a trend to lower surface tension values with increasing d. ac., but no statistically significant difference was found between the acetylated alginates. Mucoid alginate was more closely matched by the synthetically acetylated alginate with 36% d. ac.

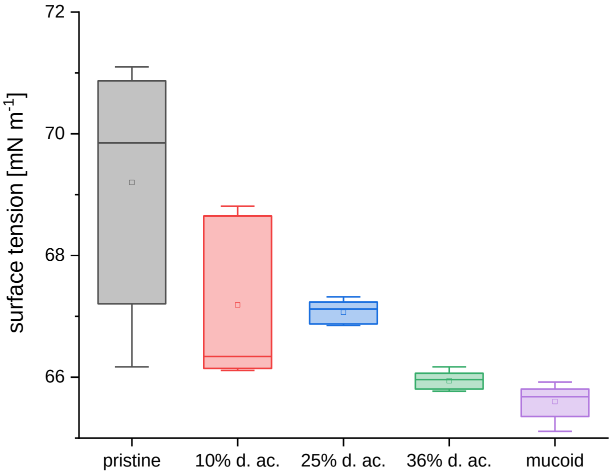

References

1. SkjÅk-Bræk, G.; Paoletti, S.; Gianferrara, T., Selective acetylation of mannuronic acid residues in calcium alginate gels. *Carbohydrate Research* **1989,** *185* (1), 119-129.
2. Pawar, S. N.; Edgar, K. J., Chemical Modification of Alginates in Organic Solvent Systems. *Biomacromolecules* **2011,** *12* (11), 4095-4103.
